# Response to “*Acute myeloneuropathy after nitrous oxide use: multimodal diagnostic insights*”

**DOI:** 10.1186/s42466-025-00432-w

**Published:** 2025-11-17

**Authors:** Julius Nicolai Meißner, Louisa Nitsch

**Affiliations:** 1 Department of Vascular Neurology, University Hospital Bonn, Venusberg Campus 1, 53127 Bonn, Germany; 2 Department of Neuroimmunology, University Hospital Bonn, Bonn, Germany

Dear Editor,

Thank you for the opportunity to respond to the correspondence entitled “*Acute Myeloneuropathy After Nitrous Oxide Use: Multimodal Diagnostic Insights*”.

With reference to our article [1] Runzeimer et al. report on diagnostic considerations in nitrous oxide induced neurological damages, presenting a typical case of a 24-year-old male patient showing subacute severe sensorimotor deficits of the lower limbs following recreational nitrous oxide use. Diagnostic workup included laboratory analyses, magnetic resonance imaging of the spinal cord and nerve conduction studies.

The report raises important points of an increasingly relevant neurological disorder. Besides typical imaging and nerve conduction findings, diagnostic pitfalls encountered in functional vitamin B_12_ deficiency are demonstrated. Especially, serum neurofilament light chain (NfL) levels have not yet been systematically studied in nitrous oxide induced neurological disorders. A relationship of methylmalonic acid levels and NfL levels has recently been demonstrated, possibly linking mitochondrial dysfunction to neuronal damage in nitrous oxide induced myeloneuropathy [2].

However, the specifity of NfL levels is limited. It remains unclear whether it helps distinguish between acute inflammatory polyradiculoneuropathy, or myelopathy of other etiologies, and nitrous oxide-induced disorders [3]. It may be more useful as a parameter for monitoring neurological recovery. Taken together, this report underscores the increasing relevance of the disorder, as it adds to several similar recent case series [4, 5] and a recent survey conducted among practicing neurologists [6].

Finally, we emphasize the call for policy reassessment regarding the accessibility of nitrous oxide.

Yours sincerely,

Julius Meißner and Louisa Nitsch.

## Data Availability

The data supporting the findings of this case report are available from the corresponding author upon reasonable request.
